# The effect of nonrestorative sleep on incident hypertension 1–2 years later among middle-aged Hispanics/Latinos

**DOI:** 10.1186/s12889-023-16368-2

**Published:** 2023-07-31

**Authors:** Kaori Saitoh, Takuya Yoshiike, Yoshiyuki Kaneko, Tomohiro Utsumi, Kentaro Matsui, Kentaro Nagao, Aoi Kawamura, Rei Otsuki, Yuichiro Otsuka, Sayaka Aritake-Okada, Yoshitaka Kaneita, Hiroshi Kadotani, Kenichi Kuriyama, Masahiro Suzuki

**Affiliations:** 1 Department of Psychiatry, Nihon University School of Medicine, 30-1 Oyaguchi-kamicho, Itabashi-ku, Tokyo, 173-8610 Japan; 2 Department of Sleep-Wake Disorders, National Institute of Mental Health, National Center of Neurology and Psychiatry, Tokyo, Japan; 3 Fondation FondaMental, Créteil, France; 4 Université Paris Est Créteil, INSERM U955, IMRB, Laboratoire Neuro-Psychiatrie translationnelle, Créteil, France; 5 Department of Psychiatry, The Jikei University School of Medicine, Tokyo, Japan; 6 Department of Clinical Laboratory, National Institute of Mental Health, National Center of Neurology and Psychiatry, Tokyo, Japan; 7 Department of Psychiatry, Tokyo Women’s Medical University, Tokyo, Japan; 8 Department of Psychiatry, National Center Hospital, National Center of Neurology and Psychiatry, Tokyo, Japan; 9 Department of Psychiatry and Behavioral Sciences, Graduate School of Medical and Dental Sciences, Tokyo Medical and Dental University, Tokyo, Japan; 10 Division of Public Health, Department of Social Medicine, Nihon University School of Medicine, Tokyo, Japan; 11 Department of Health Sciences, Saitama Prefectural University, Saitama, Japan; 12 Department of Psychiatry, Shiga University of Medical Science, Shiga, Japan

**Keywords:** Nonrestorative sleep, Incident hypertension, Insomnia, Middle-aged adults, Hispanic/Latinos, Young adults

## Abstract

**Background:**

Insomnia is known to be a major risk factor for incident hypertension. Nonrestorative sleep (NRS), which refers to insufficiently rested sleep, has reported to associate with various diseases. This study aimed to investigate the longitudinal association between insomnia-related symptoms including NRS and incident hypertension 1**–**2 years later by age group (young, 18–39 years and middle-age, 40–64 years) using existing cohort data involving Hispanics/Latinos.

**Methods:**

This study included 1100 subjects who had participated in both the Hispanic Community Health Study/Study of Latinos and its follow-up study, the Sueño Ancillary Study, and met additional eligibility criteria. Incident hypertension was assessed by self-reported history and/or the use of antihypertensives. The Women’s Health Initiative Insomnia Rating Scale (WHIIRS) was used to evaluate insomnia-related symptoms (difficulty initiating sleep, difficulty maintaining sleep, early morning awakening, difficulty returning to sleep, and NRS). Logistic regression analyses were conducted to assess the degree to which insomnia-related symptoms at baseline predicted incident hypertension.

**Results:**

Among the participants (64% middle-aged, 36% young adults), 140 (12.7%) developed hypertension during the follow-up period. Among the sleep-related symptoms, only NRS predicted incident hypertension after adjusting for sociodemographic factors and physical condition (odds ratio: 1.88, 95% confidence interval: 1.10–3.21, p = 0.022) in middle-aged adults. None of the insomnia-related symptoms were associated with incident hypertension in the young adults. No association was found between WHIIRS-defined insomnia (total score ≥ 9) and incident hypertension in middle-aged adults or young adults.

**Conclusion:**

The present findings suggest the importance of focusing on NRS to help prevent the development of hypertension in middle-aged adults.

## Background

At present, one in three adults around the world are diagnosed as having hypertension, and the number of hypertensive patients has doubled in the past 30 years [1]. Hypertension is the principal risk factor for major life-threatening diseases such as stroke and ischemic heart disease [2]. Furthermore, the incidence of hypertension increases with age [3], and the global population aged ≥ 65 years is growing faster than all other age groups. Thus, the establishment of early and efficient methods for the prevention of hypertension is urgently needed.

Diabetes, obesity, male sex, insufficient exercise, insomnia, smoking, and alcohol consumption have been reported to be risk factors for incident hypertension [4–8]. Therefore, focusing on such factors is considered useful for reducing the incidence of hypertension. However, a recent study reported that the effect of these risk factors on hypertension differ depending on age. Zang et al. reported that male sex was a significant risk factor in young and middle-aged individuals, but not in older adults. Moreover, obesity, diabetes, a family history of stroke, and hypertriglyceridemia were significant risk factors in all age groups, but the degrees of the effects differed depending on age [9]. Consequently, for more effective prevention, individual risks should be examined by age.

In addition to the above factors, insomnia is also recognized as a risk factor for hypertension. A recent meta-analysis reported that insomnia increased the risk of incident hypertension by about 1.2 times [10]. As for subtypes of insomnia symptoms (difficulty initiating sleep [DIS], difficulty maintaining sleep [DMS], and early morning awakening [EMA]), a previous study found that DIS was not associated with the development of hypertension, whereas DMS and EMA were, with 1.3- and 1.1-fold increased risks, respectively [10]. Because the incidence of insomnia increases with age [11], the significance of insomnia as a risk factor for hypertension may also vary with age. However, how each insomnia symptom relates to the development of hypertension in different generations remains unclear.

Nonrestorative sleep (NRS), which refers to sleep that does not result in feeling rested [12], was excluded from the core symptoms of insomnia in the revision of the International Classification of Sleep Disorders from the Second to the Third Edition [13]. However, NRS has recently been reported to be associated with various diseases, such as diabetes, gastroesophageal reflex disease, eye diseases, arthritis, eczema, and depression [14–16]. In addition, a prospective study reported that NRS was a risk factor for incident coronary artery heart disease (CHD) [17]. Given that hypertension is one of the major risk factors for CHD [18], NRS may be associated with the development of CHD via the develop of hypertension.

A few studies have investigated the association between NRS and hypertension, but the results are conflicting, possibly due to methodological differences [15, 19]. As mentioned earlier, the degrees of the effects of risk factors for the incidence of hypertension differ depending on age. Therefore, the effect of NRS on the development of hypertension should also be examined by age group.

Given this background, the present study aimed to investigate the longitudinal association between insomnia-related symptoms (DIS, DMS, EMA, difficulty returning to sleep [DRS], and NRS) and incident hypertension by age group (young, 18–39 years and middle-age, 40–64 years) using existing cohort data from studies involving Hispanics/Latinos living in the USA [20, 21].

## Methods

We used existing longitudinally measured cohort data from Hispanics/Latinos living in the USA taken from the Hispanic Community Health Study/Study of Latinos (HCHS/SOL) and its follow-up study conducted 1–2 years later, the Sueño Ancillary Study.

The present study was approved by the ethics committee of the National Center of Neurology and Psychiatry (A2020-012). All data analyzed in this are publicly available (sleepdata.org).

### Description of the data set

The HCHS/SOL study (Visit 1: V1) was a multicenter cohort study involving Hispanics/Latinos living in the USA conducted by the National Heart, Lung, and Blood institute and six other institutes of the National Institutes of Health from 2008 to 2010. The study was conducted at four sites to specify protective and harmful factors in regard to the health of Hispanics/Latinos: Bronx, Chicago, Miami, and San Diego. The HCHS/SOL study sample and design have been described elsewhere [22, 23]. The participants were identified and recruited for the present study using population-based sampling. The total number of participants (age range, 18–74 years) was 16,415. After the date was decided by telephone, the participants came to the field center to undergo the examination.

**Fig. 1:**
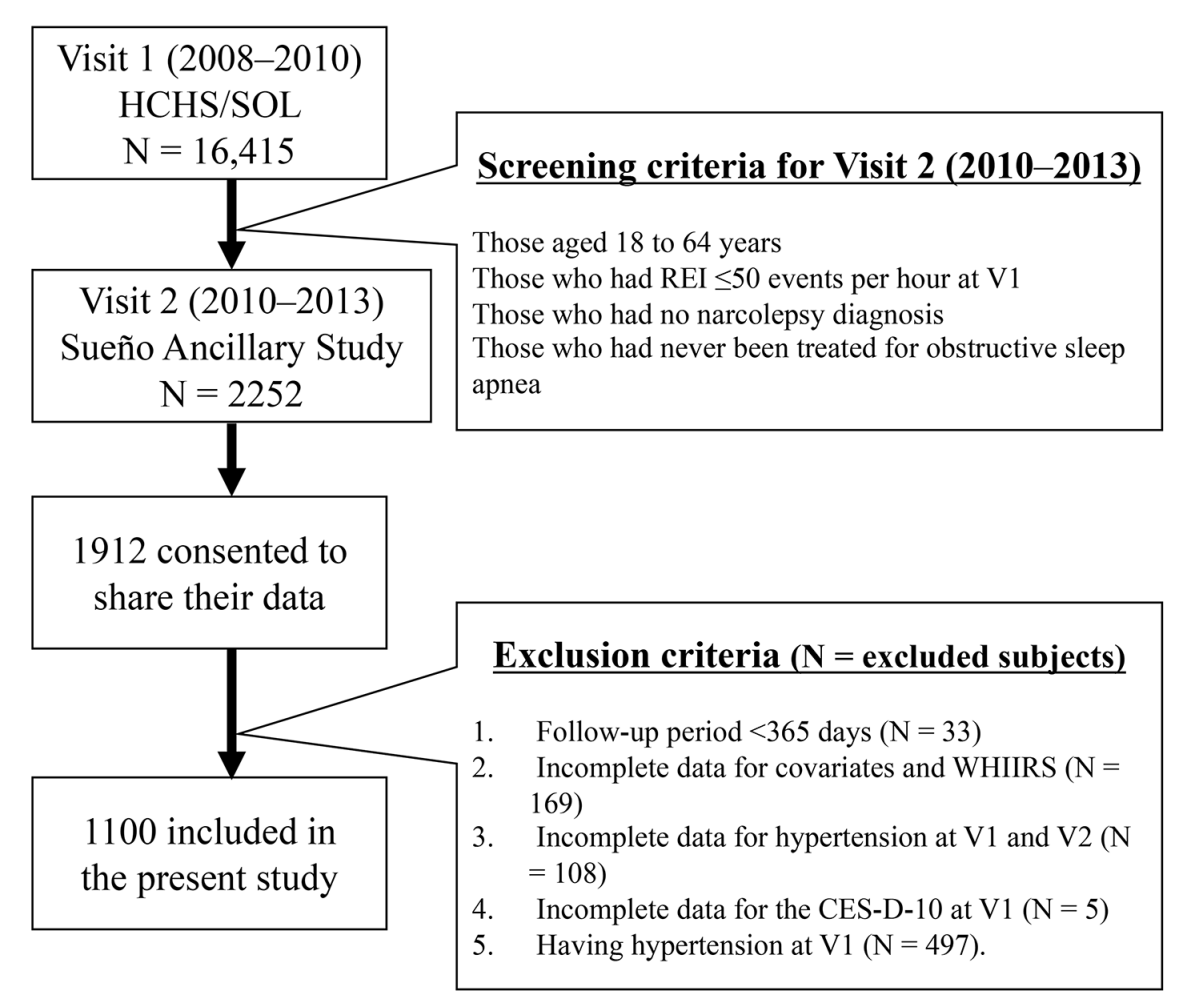
Sample flowchart HCHS/SOL: Hispanic Community Health Study/Study of Latinos, V1: Visit 1, V2: Visit 2, REI: respiratory event index, WHIIRS: Women’s Health Initiative Insomnia Rating Scale, CES-D-10: 10-item Center for Epidemiologic Studies Depression Scale

The Sueño Ancillary Study (Visit 2: V2), the follow-up study of the HCHS/SOL study, was conducted from 2010 to 2013. The inclusion criteria were as follows: age 18–64 years, the absence of severe sleep-disordered breathing (respiratory event index [REI] > 50 events per hour at the baseline examination or positive airway pressure treatment for obstructive sleep apnea [OSA]), or narcolepsy. Consequently, 2,252 individuals were selected and underwent the re-examination at each field center. Finally, 1,912 participants gave permission to researchers outside the HCHS/SOL to use their data (Fig. 1). Approval for both studies was obtained from the Institutional Review Boards at each of the participating sites. All participants provided informed consent.

### Additional criteria for the present study

The following inclusion criteria were added for the present study: (1) those who had more than 1 year of follow-up between V1 and V2; (2) those who had complete data on covariates (i.e., age, sex, participating sites, body mass index [BMI], alcohol use, cigarette use, marital status, income, education level, follow-up periods, sleep duration, Global Physical Activity Questionnaire [GPAQ], Short-Form 12-Item Survey—version 2 [SF-12v2], 10-item Center for Epidemiologic Studies Depression Scale [CES-D-10], chronic obstructive pulmonary disease [COPD], diabetes, and respiratory event index [REI]) and the Women’s Health Initiative Insomnia Rating Scale (WHIIRS) from V1; (3) those who had complete data on hypertension (questions about the diagnosis and use of antihypertensives) from both V1 and V2; (4) those who had completed the CES-D-10 at V1; and (5) those who did not have hypertension at V1. Finally, 1100 subjects were included in this study (Fig. 1).

## Measures

### Incident hypertension

The presence of hypertension was assessed using questions about the diagnosis and use of antihypertensives. At both V1 and V2, the participants were asked about a diagnosis of hypertension by using the following question: “Has a doctor ever said that you have high blood pressure or hypertension?” They were also asked to declare all prescribed or over-the-counter medications taken within the past 4 weeks at both time periods. Participants who had been diagnosed with hypertension and/or were taking antihypertensive medications at V1 were excluded. Among the other participants, those who had been diagnosed with hypertension and/or were taking antihypertensive medications at V2 were defined as those who developed hypertension during the follow-up period.

### Insomnia-related symptoms

The WHIIRS [24], the internal consistency of which has been confirmed within acceptable limits (α = 0.78) [24], was used to assess the following five insomnia-related symptoms for the previous 4 weeks:

1) DIS: “Did you have trouble falling asleep?”

2) DMS: “Did you wake up several times at night?”

3) EMA: “Did you wake up earlier than you planned to?”

4) DRS: “Did you have trouble getting back to sleep after you woke up too early?”

5) NRS: “Overall, was your typical night’s sleep during the past 4 weeks:”

For questions 1) through 4), the participants chose from the following five responses: 1, No, not in past 4 weeks; 2, Yes, less than once a week; 3, Yes, 1 or 2 times a week; 4, Yes 3 or 4 times a week; and 5, Yes, 5 or more times a week. We regarded those who answered 1 and 2 as not having the respective sleep problem, and those who answered 3–5 as having the respective sleep problem [25]. For question 5), the participants chose from the following five responses: 0, Very sound or restful; 1, Sound or restful; 2, Average quality; 3, Restless; and 4, Very restless. We defined those who answered 0–2 to the questions as having restorative sleep, and those who answered 3 and 4 as having NRS [25].

Based on a previous study [24], a WHIIRS total score of ≥ 9 was used as the cutoff for insomnia. The original response score ranges from 0 to 4, whereas the response score used in the present study ranged from 1 to 5. The total score was calculated using the following formula [16]: WHIIRS total score = DIS + DMS + EMA + DRS + NRS – 4.

### Covariates

#### Sociodemographic variables

We treated all sociodemographic data at V1, except for physical health, as categorical variables. The sociodemographic variables included as covariates were as follows: age (≤29, 30–39, 40–49, 50–59, ≥ 60 years), gender (male, female), BMI (< 25, ≥ 25 kg/m^2^), field center (Bronx, Chicago, Miami, San Diego), income (< 30,000 USD, ≥ 30,000 USD, not reported), education level (less than high school, high school, and more than high school), marital status (single, married or living with a partner, separated, divorced, or widowed), alcohol drinking (never, former, current), and smoking (never, former, current). Physical activity was assessed using the GPAQ, and was categorized into three levels (low, moderate, high) [26].

Physical health was evaluated using the SF-12v2, which provided a numeric variable score that was calculated using a standardized score of 0–100 [27].

#### Sleeping pill use

The use of sleeping pills at V1 was assessed the following question: “Did you take sleeping pills to help you sleep ?”. Participants chose from the following five responses: 1, No, not in the past 4 weeks; 2, Yes, less than once a week; 3, Yes, 1 or 2 times a week; 4, Yes, 3 or 4 times a week; and 5, Yes, 5 or more times a week. We defined those who answered 2–5 as sleeping pill users.

#### Sleep duration

Sleep duration was assessed separately for weekdays and weekends by using the following questions: “What time do you usually go to bed?” and “What time do you usually wake up?”. Average weekly sleep duration was calculated using the following formula:

Average sleep duration = ([weekend sleep duration] × 2 + [weekday sleep duration] × 5) / 7.

Sleep duration was divided into five groups (< 6 h; ≥6 to < 7 h; ≥7 to < 8 h; ≥8 to < 9 h; ≥9 h). A previous meta-analysis reported a U-shaped relationship between sleep duration and mortality [28], with those who slept approximately 7 h having the lowest risk of death. Therefore, we used those who slept ≥7 to < 8 h with the lowest health risk as a reference [29].

#### Follow-up period

We classified the follow-up period into two groups (1 year: ≥365 to ≤729 days and 2 years: ≥730 days).

### Physical and mental disorders

#### Obstructive sleep apnea (OSA)

The presence of OSA at V1 was examined using a home sleep apnea devise (ARES Unicorder 5.2; B-Alert, Carlsbad, CA). Those who had an REI: respiratory event frequency per hour of monitoring ≥ 15 were defined as having OSA.

#### Chronic obstructive pulmonary disease (COPD)

The participants who answered on a self-report respiratory form that a doctor had informed them that they had COPD or emphysema were considered to have COPD.

#### Diabetes

The presence of diabetes was assess using the following questions: “Has a doctor ever said that you have diabetes?” and “Was this during pregnancy?”. Those who answered that a doctor had told them that they had diabetes were considered to have diabetes.

#### Depression

The CES-D-10 is composed of 10 items to assess depression over the past week, with a cutoff score of 10 [30]. The validity and reliability of the CES-D-10 have been confirmed in HCHS/SOL samples [31].

As the dependent variable was insomnia-related symptoms, we excluded the item related to sleep (item 7: “My sleep was restless”) to account for confounding bias. As a result, a total of nine items (CES-D-9) were used. The total score was calculated using the following formula:

Total CES-D-9 score = SUM (items 1–6, items 8–10) × 10/9 [16].

Participants with a score of 10 or more at V1 were considered to have depression [30].

### Statistical analysis

Differences in demographic characteristics between the participants with and without incident hypertension were examined using χ^2^ and *t* tests.

We chose possible confounders for their potential association with incident hypertension based on clinical knowledge. Due to small number of case of incident hypertension in the younger adults, we used the propensity score to calculate the effect for all confounders. A logistic regression model was conducted to estimate the propensity score, in which each insomnia-related symptom was a dependent variable and the sociodemographic and health status factors (age, gender, BMI, field center, income, education level, marital status, drinking alcohol, smoking, OSA, sleep duration, sleeping pills use, follow-up period, GPAQ, SF-12v2, depression, COPD, diabetes) were independent variables. After examining the risk of each insomnia-related symptom at V1 for the development of V2 hypertension in univariate regression, each insomnia-related symptom at V1 was regressed on the incident hypertension in V2 adjusting for the propensity score of the sociodemographic and health status factors. The level of statistical significance was set at p < 0.05. All statistical analyses were performed using SPSS ver. 26.0 (IBM, New York, USA).

We defined those aged 18–39 years as young adults and those aged 40–64 years as middle-aged, referring to the 2019 NICE NG136 criteria for developing hypertension in young adults (< 40 years old) [32].

## Results

### Demographic characteristics

Of the 1100 participants, 393 and 707 were categorized as young and middle-aged adults, respectively. Tables 1 and 2 show the participants’ demographic characteristics at V1 for both age groups.

**Table 1:** Demographic data among young adults in this study

			All (N = 393)			No HT (N = 366)			HT incidence (N = 27)			Prevalence of HT			
			n	(%)		n	(%)		n	(%)				p	
HT															
	No HT		366	93.1											
	HT incidence		27	6.9											
Gender															
	Male		158	40.2		144	39.3		14	51.9		8.9%		0.201	
	Female		235	59.8		222	60.7		13	48.1		5.5%			
Age, years															
	18–29		203	51.7		192	52.2		11	40.7		5.4%		0.240	
	30–39		190	48.3		174	47.5		16	59.3		8.4%			
BMI, kg/m^2^															
	< 25		120	30.5		115	31.4		5	18.5		4.2%		0.160	
	≥25		273	69.5		251	68.6		22	81.5		8.1%			
Center															
	Bronx		98	24.9		85	23.2		13	48.1		13.3%		0.034	*
	Chicago		108	27.5		102	27.9		6	22.2		3.8%			
	Miami		132	33.6		127	34.7		5	18.5		5.5%			
	San Diego		55	14.0		52	14.2		3	11.1		5.6%			
Income, USD															
	< 30,000		230	58.5		211	57.7		19	70.4		8.3%		0.367	
	≥30,000		127	32.2		120	32.8		7	25.9		5.5%			
	Not reported		36	9.2		35	9.6		1	3.7		2.8%			
Education															
	Less than high school (HS)		101	25.7		91	24.9		10	37.0		9.9%		0.162	
	HS and more than HS		292	74.3		275	75.1		17	63.0		5.8%			
Marital status															
	Single		205	52.2		189	51.6		16	59.3		7.8%		0.691	
	Married or living with a partner		163	41.5		153	41.8		10	37.0		6.1%			
	Separated, divorced, or widowed		25	6.4		24	6.6		1	3.7		4.0%			
Alcohol use															
	Never		83	21.1		83	22.7		0	0.0		0.0%		0.018	*
	Former		126	32.1		114	31.1		12	44.4		9.5%			
	Current		184	46.8		169	46.2		15	55.6		8.2%			
Cigarette use															
	Never		275	70.0		257	70.2		18	66.7		6.5%		0.698	
	Former		39	9.9		37	10.1		2	7.4		5.1%			
	Current		79	20.1		72	19.7		7	25.9		8.9%			
Sleeping medication															
	Not, in the past 4 weeks		344	87.2		321	87.7		23	85.2		6.7%		0.702	
	Yes, in the past 4 weeks		49	14.8		45	12.3		4	14.8		8.2%			
Obstructive sleep apnea (OSA)															
	OSA+		5	1.3		3	0.8		2	7.4		40.0%		0.003	**
	OSA–		388	98.7		363	99.2		25	92.6		6.0%			
Follow-up															
	1 year		127	32.3		123	33.6		4	14.8		3.1%		0.044	*
	2 years		266	67.7		243	66.4		23	85.2		8.6%			
Sleep duration, hours															
	< 6		19	4.8		19	5.2		0	0.0		0.0%		0.185	
	≥6 to 7		55	14.0		51	13.9		4	14.8		7.3%			
	≥7 to 8		111	28.2		107	29.2		4	14.8		3.6%			
	≥8 to 9		109	27.7		97	26.5		12	44.4		11.0%			
	≥9		99	25.2		92	25.1		7	25.9		7.1%			
GPAQ															
	Low		158	40.2		148	40.4		10	37.0		6.3%		0.255	
	Moderate		186	47.3		170	46.4		16	59.3		8.6%			
	High		49	12.5		48	13.1		1	3.7		2.0%			
Physical health (SF-12v2)						53 ± 6.7			49.4 ± 7.6					0.010	*
Depression															
	No		293	74.6		279	76.2		14	51.9		4.8%		0.005	**
	Yes		100	25.4		87	23.8		13	48.1		13.0%			
COPD															
	No		391	99.5		364	99.5		27	100		6.9%		0.700	
	Yes		2	0.5		2	0.5		0	0		0.0%			
Diabetes															
	No		380	96.7		356	97.3		24	88.9		6.3%		0.019	*
	Yes		13	3.3		10	2.7		3	11.1		23.1%			

*p < 0.05, **p < 0.01

HT: hypertension, BMI: body mass index, GPAQ: Global Physical Activity Questionnaire, SF-12v2: Short-Form 12-Item Survey—version 2, COPD: chronic obstructive pulmonary disease

**Table 2:** Demographic data among middle-aged adults in this study

			All (N = 707)			No HT (N = 594)			HT incidence (N = 113)			Prevalence of HT			
			n	(%)		n	(%)		n	(%)				p	
HT															
	No HT		594	84.0											
	HT incidence		113	16.0											
Gender															
	Male		236	33.4		197	33.2		39	34.5		16.5%		0.781	
	Female		471	66.6		397	66.8		74	65.5		15.7%			
Age, years															
	40–49		383	54.2		327	55.1		56	49.6		14.6%		0.007	**
	50–59		260	36.8		222	37.4		38	33.6		14.6%			
	60–65		64	9.1		45	7.6		19	16.8		29.7%			
BMI, kg/m^2^															
	< 25		141	19.9		127	21.4		14	12.4		9.9%		0.028	*
	≥25		566	80.1		467	78.6		99	87.6		17.5%			
Center															
	Bronx		190	26.9		156	26.3		34	30.1		17.9%		0.258	
	Chicago		170	24.0		148	24.9		22	19.5		14.5%			
	Miami		242	34.2		207	34.8		35	31.0		21.0%			
	San Diego		105	14.9		83	14.0		22	19.5		12.9%			
Income, USD															
	< 30,000		484	68.5		402	67.7		82	72.6		16.9%		0.329	
	≥30,000		200	28.3		174	29.3		26	23.0		13.0%			
	Not reported		23	3.3		18	3.0		5	4.4		21.7%			
Education															
	Less than high school (HS)		226	32.0		187	31.5		39	34.5		17.3%		0.526	
	HS and more than HS		481	68.0		407	68.5		74	65.5		15.4%			
Marital status															
	Single		150	21.2		128	21.5		22	19.5		14.7%		0.615	
	Married or living with a partner		388	54.9		328	55.2		60	53.1		15.5%			
	Separated, divorced, or widowed		169	23.9		138	23.2		31	27.4		18.3%			
Alcohol use															
	Never		145	20.5		117	19.7		28	24.8		19.3%		0.470	
	Former		220	31.1		187	31.5		33	29.2		15.0%			
	Current		342	48.4		290	48.8		52	46		15.2%			
Cigarette use															
	Never		405	57.3		344	57.9		61	54		15.1%		0.734	
	Former		142	20.1		118	19.9		24	21.2		16.9%			
	Current		160	22.6		132	22.2		28	24.8		17.5%			
Sleeping medication															
	Not, in the past 4 weeks		597	84.4		503	84.7		94	83.2		15.7%		0.688	
	Yes, in the past 4 weeks		110	15.6		91	15.3		19	16.8		17.3%			
Obstructive sleep apnea (OSA)															
	OSA+		54	7.6		46	7.7		8	7.1		15.0%		0.807	
	OSA–		653	92.4		548	92.3		105	92.9		16.0%			
Follow-up															
	1 year		222	31.4		184	31.0		38	33.6		17.1%		0.578	
	2 years		485	68.6		410	69.0		75	66.4		15.5%			
Sleep duration, hours															
	< 6		54	7.6		40	6.7		14	12.4		25.9%		0.163	
	≥6 to 7		118	16.7		102	17.2		16	14.2		13.6%			
	≥7 to 8		233	33		200	33.7		33	29.2		14.2%			
	≥8 to 9		177	25		144	24.2		33	29.2		18.6%			
	≥9		125	17.7		108	18.2		17	15.0		13.6%			
GPAQ															
	Low		345	48.8		289	48.7		56	49.6		16.2%		0.922	
	Moderate		312	44.1		262	44.1		50	44.2		16.0%			
	High		50	7.1		43	7.2		7	6.2		14.0%			
Physical health (SF-12v2)						49.7 ± 9.3			48.7 ± 9.8					0.314	
Depression															
	No		493	69.7		421	70.9		72	63.7		14.6%		0.129	
	Yes		214	30.3		173	29.1		41	36.3		19.2%			
COPD															
	No		697	98.6		585	98.5		112	99.1		16.1%		0.603	
	Yes		10	1.4		9	1.5		1	0.9		10.0%			
Diabetes															
	No		600	84.9		510	85.9		90	79.6		15.0%		0.091	
	Yes		107	15.1		84	14.1		23	20.4		21.5%			

*p < 0.05, **p < 0.01

HT: hypertension, BMI: body mass index, GPAQ: Global Physical Activity Questionnaire, SF-12v2: Short-Form 12-Item Survey—version 2, COPD: chronic obstructive pulmonary disease

The mean age of the young adults, who were predominantly female, was 28.7 ± 7.0 years. 70% of the sample reported having obesity and being a nonsmoker. More than half of the individuals had a household income < 30,000 USD, an education history of high school or more than high school, were single, and currently consumed alcohol. Most of the individuals had not used sleeping pills in the past 4 weeks, did not have OSA or COPD, and had low or moderate physical activity. The mean sleep duration was 8.1 ± 1.4 h. About 25% of the individuals had depressive symptoms, and 3% reported diabetes.

The mean age of the middle-aged adults, who were also predominantly female, was 49.8 ± 6.1 years. 80% of the sample reported having obesity. More than half of the individuals had a household income < 30,000 USD, an education history of high school or more than high school, were married or living with a partner, and had consumed alcohol. 70% were nonsmokers. Most of individuals had not used sleeping pills in the past 4 weeks, did not have OSA or COPD, and had low or moderate physical activity. The mean sleep duration was 7.7 ± 1.4 h. Almost 30% of the individuals had depressive symptoms and 15% reported diabetes.

### Incident hypertension

The average follow-up period was 786 ± 135 days. Of 1100 participants included in the analysis, 140 (12.7%) had incident hypertension at V2. No significant difference in incident hypertension was found by gender (male vs. female: 13.5% vs. 12.3%, χ^2^ = 0.29, df = 1, p = 0.59). In addition, the proportion of those who developed hypertension increased with age (χ^2^ = 31.58, df = 4, p < 0.001), incident hypertension was almost twice as high in those aged 40–59 as in those aged 18–39 years, and was twice as high in those aged 60–64 as in those aged 40–59 years (Table 3). The prevalence of incident hypertension was 6.9% among young and 16.0% among middle-aged adults.

**Table 3:** Incident hypertension by age group

		No HT			HT incidence			Prevalence of HT	
		N	(%)		N	(%)		(%)	
All	Age, years	960			140				
	18–29	192	20.0		11	7.9		5.4	
	30–39	174	18.1		16	11.4		8.4	
	40–49	327	34.1		56	40.0		14.6	
	50–59	222	23.1		38	27.1		14.6	
	60–64	45	4.7		19	13.6		29.7	

HT: hypertension

### Prevalence of insomnia/insomnia-related symptoms

The young adults had a significantly lower prevalence of insomnia and WHIIRS scores compared with the middle-age adults (young vs. middle-aged, prevalence of insomnia: 27.0% vs. 37.6%, χ^2^ = 12.8, df = 1, p < 0.001, WHIIRS: 5.9 ± 4.9 vs. 7.2 ± 5.5, *t* (887) = − 3.97, p < 0.001). Table 4 shows the results regarding the prevalence of insomnia and the five insomnia-related symptoms by gender and age. Females had a significantly higher prevalence of insomnia and all five symptoms compared with males (insomnia: χ^2^ = 12, df = 1, p < 0.001, DIS: χ^2^ = 10, df = 1, p = 0.002, DMS: χ^2^ = 5.9, df = 1, p = 0.015, EMA: χ^2^ = 5.3, df = 1, p = 0.021, DRS: χ^2^ = 10, df = 1, p = 0.002, NRS: χ^2^ = 10, df = 1, p = 0.001).

**Table 4:** Prevalence of each of the five insomnia-related symptoms by sex and age

		Insomnia		DIS		DMS		EMA		DRS		NRS
All												
	18–29	21%		36%		41%		32%		24%		13%
	30–39	33%		36%		51%		40%		31%		14%
	40–49	33%		38%		53%		43%		32%		16%
	50–59	42%		48%		64%		49%		42%		16%
	60–64	48%		50%		64%		50%		48%		22%
	Total	34%		40%		54%		42%		34%		15%
Males												
	18–29	21%		39%		36%		33%		25%		14%
	30–39	35%		41%		52%		38%		29%		9%
	40–49	24%		28%		45%		43%		25%		7%
	50–59	33%		35%		64%		36%		31%		13%
	60–64	29%		24%		57%		24%		33%		14%
	Total	27%		34%		49%		37%		27%		11%
Females												
	18–29	22%		32%		45%		31%		23%		12%
	30–39	32%		34%		51%		40%		32%		17%
	40–49	38%		43%		57%		43%		35%		20%
	50–59	46%		54%		64%		55%		47%		17%
	60–64	58%		63%		67%		63%		56%		26%
	Total	38%		44%		56%		45%		37%		18%

DIS: difficulty initiating sleep, DMS: difficulty maintaining sleep, EMS: early morning awakening, DRS: difficulty returning to sleep, NRS: nonrestorative sleep

In all subjects, the prevalences of insomnia (χ^2^ = 27.6, df = 4, p < 0.001), DIS (χ^2^ = 12.4, df = 4, p = 0.015), DMS (χ^2^ = 27.5, df = 4, p < 0.001), EMA (χ^2^ = 15.9, df = 4, p = 0.003), and DRS (χ^2^ = 24.1, df = 4, p < 0.001) tended to increase with advanced age. However, no such increase with age was observed for NRS.

### Association between insomnia/insomnia-related symptoms and incident hypertension

Table 5 shows the results of logistic regression analysis for the association between insomnia/insomnia-related symptoms and incident hypertension. Neither insomnia nor any insomnia-related symptoms was associated with incident hypertension in univariate logistic analysis among the young adults.

**Table 5:** Association between insomnia-related symptoms and incident hypertension

		Crude OR (95% CI)			Adjusted OR (95% CI)		
			p			p	
< 40 (27/393)							
Insomnia		1.7 (0.7–3.7)	0.226		1.3 (0.5–3.4)	0.525	
DIS		0.7 (0.3–1.7)	0.484		0.6 (0.2–1.5)	0.258	
DMS		2.1 (0.9–4.7)	0.069		1.7 (0.7–3.9)	0.237	
EMA		1.5 (0.7–3.3)	0.309		1.2 (0.5–2.9)	0.651	
DRS		1.6 (0.7–3.7)	0.239		1.3 (0.5–3.1)	0.553	
NRS		1.1 (0.4–3.4)	0.834		1.2 (0.4–3.7)	0.814	
≥40 (113/707)							
Insomnia		1.6 (1.1–2.5)	0.016	*	1.4 (0.9–2.2)	0.159	
DIS		1.4 (0.9–2.1)	0.110		1.2 (0.7–1.8)	0.499	
DMS		1.6 (1.0–2.4)	0.030	*	1.4 (0.9–2.2)	0.138	
EMA		1.4 (0.9–2.1)	0.121		1.2 (0.8–1.8)	0.449	
DRS		1.4 (0.9–2.1)	0.122		1.1 (0.7–1.7)	0.647	
NRS		2.0 (1.3–3.3)	0.004	**	1.9 (1.1–3.2)	0.022	*

*p < 0.05, **p < 0.01

OR: odds ratio, CI: confidence interval, DIS: difficulty initiating sleep, DMS: difficulty maintaining sleep, EMA: early morning awakening, DRS: difficulty returning to sleep, NRS: nonrestorative sleep

Adjusted the each propensity score

On the other hand, among middle-aged adults, significant associations were found between incident hypertension and insomnia, DIS and NRS and (insomnia, odds ratio [OR] = 1.65; DMS, OR = 1.60; NRS, OR = 2.04) in univariate logistic regression analysis. After adjusting for the propensity scores of sociodemographic factors and health status factors, only NRS showed a significant association with incident hypertension (OR: 1.88, 95% confidence interval: 1.10–3.21, p = 0.022).

## Discussion

The results of the present study indicated age differences in the association between insomnia-related symptoms and incident hypertension. In middle-aged adults (40–64 years), multivariate analysis revealed that among the insomnia-related symptoms, only NRS was a significant predictor of incident hypertension at 1–2 years later. Conversely, in young adults (18–39 years), none of the insomnia-related symptoms were associated with incident hypertension at 1–2 years later. No association was found between WHIIRS-defined insomnia (total score ≥ 9) and incident hypertension in middle-aged adults or young adults.

To the best of our knowledge, this is the first study to investigate the longitudinal association between NRS and incident hypertension by age group. Our findings suggest the importance of focusing on NRS in addition to other insomnia symptoms to help prevent the development of hypertension in middle-aged adults.

### Incident hypertension

The rate of incident hypertension in this study was 12.7%. Previous studies investigating the association between insomnia and incident hypertension in the USA reported that the rate of incident hypertension ranged from 5.4–36.0% [33–35]. This wide range of incidence might be due to methodological differences such as observational periods. Given that the follow-up period in the present study was approximately 2 years, the incidence of hypertension was comparable to those of previous studies. However, the Hispanic Latinos living in the USA are characterized by lower rates of disease awareness compared with non-Hispanic whites [36]. Therefore, the actual incidence of hypertension might be higher.

### Prevalence of insomnia/insomnia-related symptoms

The prevalence of insomnia, DIS, DMS, and EMA were 33.8%, 40.3%, 53.6%, and 41.9%, respectively. The prevalence of insomnia and DMS were similar to those reported in previous general adult population surveys, but the prevalence of DIS and EMA were higher than those of the general population [37]. This may be due to differences in the participants’ age and gender. Furthermore, we also found that the prevalence of these core symptoms of insomnia increased with age and were significantly higher in women than in men; these findings were also in agreement with a previous study [37].

The prevalence of NRS in the present study was 15.3%, which was comparable with that reported by a study involving a large European population (10.8%) [38]. The present results are consistent with that study in that NRS was significantly more common in females than in males [38]. No age differences were found with regard to the prevalence of NRS, but a previous study reported that the prevalence of NRS tended to be higher in a younger age group [38]. On the other hand, another study reported finding a higher prevalence of NRS in middle-aged and older adults [39]. Therefore, no agreement has been obtained with regard to the age-related prevalence of NRS.

### Association between insomnia/insomnia-related symptoms and incident hypertension

In the present study, no significant association was found between insomnia/insomnia-related symptoms and incident hypertension in young adults. Young adults tend to complain of insomnia-related symptoms less frequently than do middle-aged adults [40]. Therefore, factors other than insomnia-related symptoms may have affected the prevalence of incident hypertension in this age group. Indeed, based on a comparison of demographic data between young adults with and without hypertension (Table 2), differences were found in some factors, including alcohol consumption, OSA, depressive symptoms, and diabetes. These risk factors may have contributed to incident hypertension more than to insomnia-related symptoms. In addition, the small sample size of young adults in this study may have reduced the statistical power.

Although NRS was a predictor of incident hypertension in middle-aged adults, insomnia and other sleep-related symptoms were not. Some general population studies targeting similar age groups have reported inconsistent findings. A study involving military personnel reported that insomnia was associated with incident hypertension [41]. The discrepancy of these results might be explained by whether chorionic insomnia was investigated. Cheng et al. [33] investigated the association between the core symptoms of insomnia (DIS and DMS) and the onset of hypertension 1 year later in those aged in their 30 to 50 s, and reported that DMS was a significant risk factor of incident hypertension. Rod et al. [42] investigated these relationships over a longer follow-up period (19 years), and reported that DMS and EMA were significant predictors of incident hypertension in females and males, respectively. However, several methodological differences should be noted between our study and previous studies. Cheng et al. [33] and Rod et al. [42] evaluated the symptoms of insomnia using items different from those used in the present study and determined the presence of incident hypertension based only on the presence of hypertension, not the presence or absence of antihypertensive medication, in a questionnaire survey. In addition, some differences in terms of covariates and the prevalence of incident hypertension may have contributed to the discrepancies in the results.

### NRS as a predictor of incident hypertension among middle-aged adults

The results of the present study indicated that NRS was a predictor of incident hypertension in middle-aged adults. It is generally considered that the ability to maintain homeostasis in response to physical load is superior in younger adults than in middle-aged adults, and thus the association between NRS and the development of hypertension may be found only in middle-aged adults. Although the present results are consistent with those of another study involving middle-aged and older adults [19], an additional study involving only middle-aged adults showed discordant results [15]. The latter study included participants with persistent hypertension; that is, they did not exclude participants who had hypertension at baseline. This methodological difference might be associated with the inconsistency with our results.

Although it remains unclear why only NRS predicted incident hypertension at 1–2 years later in middle-aged adults, the following explanations might be adequate. Daytime dysfunction was considered to be profoundly associated with NRS. Ohayon et al. [38] mentioned that compared with those with the core symptoms of insomnia, subjects with NRS more often experienced a more depressive mood, anxious mood, physical fatigue, decreased efficacy, memory problems, and excessive daytime sleepiness, and consulted a physician about their sleep problems more frequently. Therefore, NRS has a close relationship with mental and physical health problems and daytime impairments, and could reflect restorative dysfunctions of the physical and psychological systems more than the core symptoms of insomnia, such as DIS, DMS, and EMA.

The present results might also be explained by the link between NRS and inflammation. Zhang et al. [43] compared C-reactive protein (CRP) in blood among four groups—subjects without NRS or insomnia, those with only NRS, those with only insomnia, and those with NRS and insomnia—and found that those with NRS and with both NRS and insomnia had significantly higher CRP levels compared with those without NRS or insomnia. It has been considered that increased CRP levels are associated with future incident hypertension and arterial stiffness [44, 45]. Therefore, NRS could lead to the development of hypertension via microinflammation. Furthermore, Mathews et al. reported decreased delta power during NREM periods in females who developed hypertension [46]. NRS has also been associated with increased alpha waves during NREM periods [47]. The association between NRS and the development of hypertension in middle-aged adults may be mediated by changes in NREM periods. Finally, NRS has been reported to be more prevalent in females [38] and almost 70% of the middle-aged subjects in this study were females. Although menopausal symptoms may underpin the significant association between NRS and the development of hypertension in middle-aged women [48], menopausal status was not evaluated in this study. Further studies using inflammatory markers and evaluations such as polysomnography and menopausal status are needed to confirm these associations.

### Limitations

The strengths of this study were the longitudinal design, division of participants into age groups, and the consideration of 18 potential confounders. However, this study had also several limitations. First, the sample size was small and no older adults were included in this study. Moreover, the sample sizes of young and middle-aged adults differed. Validation studies with a larger sample size and wider age range are needed. Second, a self-report questionnaire was used to assess hypertension and the use of antihypertensive medications. Objective assessments of hypertension such as blood pressure measurements or a review of medical records are needed for the more accurate detection of incident hypertension. Third, since the definition of NRS differed among previous studies, it was difficult to compare it with previous studies. A unified definition of NRS will be needed in the future. Fourth, this study examined with only Hispanics/Latinos living in the USA. To generalize the results of the present study, it remains necessary to confirm whether similar results can be obtained in more diverse populations. Fifth, hypertension was measured using questionnaires about hypertension and the use of antihypertensive medications that were not graded. Therefore, the effect of insomnia-related symptoms on the severity of hypertension remains unclear. Finally, the covariates did not include menopausal status or a family history of cardiovascular disease or stroke. As we did not fully adjust for these factors, and they might have affected the accuracy of the results.

## Conclusion

None of the insomnia-related symptoms examined in the present study were associated with incident hypertension among young adults. By contrast, NRS was a predictor of the development of hypertension in middle-aged adults. These findings suggest the importance of focusing on NRS in addition to other insomnia symptoms to help prevent the development of hypertension in middle-aged adults.

## Data Availability

All data analyzed in this are publicly available (sleepdata.org).

## Abbreviations

NRS: Nonrestorative sleep
HT: hypertension
DIS: difficulty initiating sleep
DMS: difficulty maintaining sleep
EMA: early morning awaking
DRS: difficulty returning to sleep
CHD: coronary artery heart disease
HCHS/SOL: the Hispanic Community Health Study/Study of Latinos
V1: Visit 1
V2: Visit 2
REI: respiratory event index
OSA: obstructive sleep apnea
BMI: body mass index
GPAQ: Global Physical Activity Questionnaire
SF-12v2: Short-Form 12-Item Survey—version 2
CES-D-10: 10-item Center for Epidemiologic Studies Depression Scale
COPD: chronic obstructive pulmonary disease
WHIIRS: the Women’s Health Initiative Insomnia Rating Scale
OR: odds ratio
CI: confidence interval
NREM: non-rapid eye movement sleep

## References

[1] Worldwide trends in (2021). Hypertension prevalence and progress in treatment and control from 1990 to 2019: a pooled analysis of 1201 population-representative studies with 104 million participants. Lancet.

[2] Lawes CM, Vander Hoorn S, Rodgers A (2008). Global burden of blood-pressure-related disease, 2001. Lancet.

[3] He J, Whelton PK (1997). Epidemiology and prevention of hypertension. Med Clin North Am.

[4] Fryar CD, Ostchega Y, Hales CM, Zhang G, Kruszon-Moran D. Hypertension prevalence and control among adults: United States, 2015–2016. NCHS Data Brief. 2017(289):1–8. 29155682

[5] Fernandez-Mendoza J, Vgontzas AN, Liao D, Shaffer ML, Vela-Bueno A, Basta M (2012). Insomnia with objective short sleep duration and incident hypertension: the Penn State Cohort. Hypertension.

[6] Schroeder EC, DuBois L, Sadowsky M, Hilgenkamp TIM (2020). Hypertension in adults with intellectual disability: prevalence and risk factors. Am J Prev Med.

[7] Henry P, Thomas F, Benetos A, Guize L (2002). Impaired fasting glucose, blood pressure and cardiovascular disease mortality. Hypertension.

[8] Jordan J, Kurschat C, Reuter H (2018). Arterial hypertension. Dtsch Arztebl Int.

[9] Zhang Y, Yang H, Ren M, Wang R, Zhao F, Liu T (2021). Distribution of risk factors of hypertension patients in different age groups in Tianjin. BMC Public Health.

[10] Li L, Gan Y, Zhou X, Jiang H, Zhao Y, Tian Q (2021). Insomnia and the risk of hypertension: a meta-analysis of prospective cohort studies. Sleep Med Rev.

[11] Morin CM, LeBlanc M, Daley M, Gregoire JP, Mérette C (2006). Epidemiology of insomnia: prevalence, self-help treatments, consultations, and determinants of help-seeking behaviors. Sleep Med.

[12] Stone KC, Taylor DJ, McCrae CS, Kalsekar A, Lichstein KL (2008). Nonrestorative sleep. Sleep Med Rev.

[13] American Academy of Sleep Medicine (2014). International classification of Sleep Disorders.

[14] Okamoto M, Kobayashi Y, Nakamura F, Musha T (2017). Association between Nonrestorative Sleep and risk of diabetes: a cross-sectional study. Behav Sleep Med.

[15] Zhang J, Lam SP, Li SX, Li AM, Wing YK (2012). The longitudinal course and impact of non-restorative sleep: a five-year community-based follow-up study. Sleep Med.

[16] Saitoh K, Yoshiike T, Kaneko Y, Utsumi T, Matsui K, Nagao K (2022). Associations of nonrestorative sleep and insomnia symptoms with incident depressive symptoms over 1–2 years: longitudinal results from the Hispanic Community Health Study/Study of Latinos and Sueño Ancillary Study. Depress Anxiety.

[17] Leineweber C, Kecklund G, Janszky I, Akerstedt T, Orth-Gomér K (2003). Poor sleep increases the prospective risk for recurrent events in middle-aged women with coronary disease. The Stockholm Female Coronary Risk Study. J Psychosom Res.

[18] Turin TC, Okamura T, Afzal AR, Rumana N, Watanabe M, Higashiyama A (2016). Impact of hypertension on the lifetime risk of coronary heart disease. Hypertens Res.

[19] Otsuka Y, Kaneita Y, Tanaka K, Itani O, Kaneko Y, Suzuki M (2023). Nonrestorative sleep is a risk factor for metabolic syndrome in the general japanese population. Diabetol Metab Syndr.

[20] Zhang GQ, Cui L, Mueller R, Tao S, Kim M, Rueschman M (2018). The National Sleep Research Resource: towards a sleep data commons. J Am Med Inform Assoc.

[21] Redline S, Sotres-Alvarez D, Loredo J, Hall M, Patel SR, Ramos A (2014). Sleep-disordered breathing in Hispanic/Latino individuals of diverse backgrounds. The Hispanic Community Health Study/Study of Latinos. Am J Respir Crit Care Med.

[22] Sorlie PD, Avilés-Santa LM, Wassertheil-Smoller S, Kaplan RC, Daviglus ML, Giachello AL (2010). Design and implementation of the Hispanic Community Health Study/Study of Latinos. Ann Epidemiol.

[23] LaVange LM, Kalsbeek WD, Sorlie PD, Avilés-Santa LM, Kaplan RC, Barnhart J (2010). Sample Design and Cohort Selection in the Hispanic Community Health Study/Study of Latinos. Ann Epidemiol.

[24] Levine DW, Kripke DF, Kaplan RM, Lewis MA, Naughton MJ, Bowen DJ (2003). Reliability and validity of the women’s Health Initiative Insomnia Rating Scale. Psychol Assess.

[25] Carroll JE, Irwin MR, Levine M, Seeman TE, Absher D, Assimes T (2017). Epigenetic aging and Immune Senescence in Women with insomnia symptoms: findings from the women’s Health Initiative Study. Biol Psychiatry.

[26] World Health Organization.Global Physical Activity Questionnaire (GPAQ). Analysis Guide Geneva, Switzerland: World Health Organization; [cited 2022 August 16]. Available from: https://cdn.who.int/media/docs/default-source/ncds/ncd-surveillance/gpaq-analysis-guide.pdf?sfvrsn=1e83d571_2.

[27] Ware, J. E., Kosinski, M., Turner-Bowker, D. M., Gandek, B. How to score version 2 of the SF-12® health survey (with a supplement documenting version 1). Lincoln, RI: QualityMetric Incorporated. 2002.

[28] Cappuccio FP, D’Elia L, Strazzullo P, Miller MA (2010). Sleep duration and all-cause mortality: a systematic review and meta-analysis of prospective studies. Sleep.

[29] Amagai Y, Ishikawa S, Gotoh T, Doi Y, Kayaba K, Nakamura Y (2004). Sleep duration and mortality in Japan: the Jichi Medical School Cohort Study. J Epidemiol.

[30] Andresen EM, Malmgren JA, Carter WB, Patrick DL (1994). Screening for depression in well older adults: evaluation of a short form of the CES-D (center for epidemiologic Studies Depression Scale). Am J Prev Med.

[31] González P, Nuñez A, Merz E, Brintz C, Weitzman O, Navas EL (2017). Measurement properties of the Center for epidemiologic Studies Depression Scale (CES-D 10): findings from HCHS/SOL. Psychol Assess.

[32] National Institute for Health and Care Excellence. 2019 [Available from: https://www.nice.org.uk/guidance/ng136.

[33] Cheng P, Pillai V, Mengel H, Roth T, Drake CL (2015). Sleep maintenance difficulties in insomnia are associated with increased incidence of hypertension. Sleep Health.

[34] Phillips B, Bůzková P, Enright P (2009). Insomnia did not predict incident hypertension in older adults in the cardiovascular health study. Sleep.

[35] Phillips B, Mannino DM (2007). Do insomnia complaints cause hypertension or cardiovascular disease?. J Clin Sleep Med.

[36] Sorlie PD, Allison MA, Avilés-Santa ML, Cai J, Daviglus ML, Howard AG (2014). Prevalence of hypertension, awareness, treatment, and control in the Hispanic Community Health Study/Study of Latinos. Am J Hypertens.

[37] Hartz AJ, Daly JM, Kohatsu ND, Stromquist AM, Jogerst GJ, Kukoyi OA (2007). Risk factors for Insomnia in a Rural Population. Ann Epidemiol.

[38] Ohayon MM (2005). Prevalence and correlates of nonrestorative sleep complaints. Arch Intern Med.

[39] Ohayon MM, Partinen M (2002). Insomnia and global sleep dissatisfaction in Finland. J Sleep Res.

[40] Ohayon M (1996). Epidemiological study on insomnia in the general population. Sleep.

[41] Lewis PE, Emasealu OV, Rohrbeck P, Hu Z (2014). Risk of type II diabetes and hypertension associated with chronic insomnia among active component, U.S. Armed Forces, 1998–2013. Msmr.

[42] Rod NH, Vahtera J, Westerlund H, Kivimaki M, Zins M, Goldberg M (2011). Sleep disturbances and cause-specific mortality: results from the GAZEL cohort study. Am J Epidemiol.

[43] Zhang J, Lamers F, Hickie IB, He JP, Feig E, Merikangas KR (2013). Differentiating nonrestorative sleep from nocturnal insomnia symptoms: demographic, clinical, inflammatory, and functional correlates. Sleep.

[44] Cheung BM, Ong KL, Tso AW, Leung RY, Xu A, Cherny SS (2012). C-reactive protein as a predictor of hypertension in the Hong Kong Cardiovascular risk factor prevalence study (CRISPS) cohort. J Hum Hypertens.

[45] Yasmin McEnieryCM, Wallace S, Mackenzie IS, Cockcroft JR, Wilkinson IB (2004). C-reactive protein is associated with arterial stiffness in apparently healthy individuals. Arterioscler Thromb Vasc Biol.

[46] Matthews KA, Chang Y, Kravitz HM, Bromberger JT, Owens JF, Buysse DJ (2014). Sleep and risk for high blood pressure and hypertension in midlife women: the SWAN (study of women’s Health across the Nation) Sleep Study. Sleep Med.

[47] Branco J, Atalaia A, Paiva T (1994). Sleep cycles and alpha-delta sleep in fibromyalgia syndrome. J Rheumatol.

[48] Reckelhoff JF (2001). Gender differences in the regulation of blood pressure. Hypertension.

